# Delays reduce culprit-presence detection but do not affect guessing-based selection in response to lineups

**DOI:** 10.1038/s41598-025-13937-w

**Published:** 2025-08-04

**Authors:** Amelie Therre, Raoul Bell, Nicola Marie Menne, Carolin Mayer, Ulla Lichtenhagen, Axel Buchner

**Affiliations:** Department of Experimental Psychology, Heinrich Heine University, Universitätsstraße 1, 40225 Düsseldorf, Germany

**Keywords:** Police lineups, Eyewitness identification, Two-high threshold eyewitness identification model, Delay, Multinomial processing tree model, Psychology, Human behaviour

## Abstract

Police lineups are conducted with varying delays between the crime and the lineup. Crime-to-lineup delays may adversely affect the detection of the presence and absence of the culprit in the lineup and may potentially affect guessing-based selection. In the present study we examined how these processes change across four crime-to-lineup delays. Participants viewed a staged-crime video and then completed simultaneous photo lineups after no delay or after a delay of one day, one week or one month. The results showed a significant decline in the probability of culprit-presence detection. The form of the decline is best described by a power function with the most rapid decline occurring at short crime-to-lineup delays. Eyewitnesses did not compensate the decline in culprit-presence detection by increasing guessing-based selection, as demonstrated by the fact that the probability of guessing-based selection remained constant across crime-to-lineup delays. The findings underscore the critical importance of conducting lineups as soon as possible after a crime to maximize the probability of memory-based-culprit detection.

## Introduction

Responses made by eyewitnesses during police lineups can serve as important evidence in criminal prosecutions^1^. In a lineup, an eyewitness sees a single suspect (who may be guilty or innocent) along with a number of fillers who are known to be innocent. The ability of an eyewitness to detect the presence of the culprit in a given culprit-present lineup or to detect the absence of the culprit in a given culprit-absent lineup depends on the eyewitness’ memory. One of the most well-established facts about memory is that it decreases with time^2–4^. Naturally, memory for crime-related details is no exception. For instance, the accuracy of eyewitness responses to crime-related questions has been found to decline with increasing delay between the crime and the interview^5–8^. This may also affect performance in a lineup given that participants’ recall of facial characteristics has been reported to decline significantly after a crime-to-lineup delay ranging from one week^9^ to three weeks^10^ and one month^11^. Although conflicting results have been reported^12^, there is a general trend towards progressively worse accuracy of face memory as a function of an increasing delay^13–18^. Therefore, it may be a concern that lineups can occur with considerable delays due to factors outside of the control of investigators, such as the time needed to identify a suspect and the availability of eyewitnesses. Furthermore, there is good reason for conducting a lineup only after a thorough investigation because this increases the probability that the actual culprit and not an innocent suspect is in the lineup^19^. Delays can also result from resource limitations, as investigators must prioritize different tasks across multiple cases. Archive studies from Great Britain on real-world lineups show that delays between the crime and the lineup range from zero days to nine years^20^, with the most frequently reported delays being one to three months^20–23^. Against this background, the present study serves to test how increasing crime-to-lineup delays affect the detection of the presence or absence of the culprit and guessing-based selection in lineups.

Particularly relevant in this context are studies that address the question of how a crime-to-lineup delay affect eyewitness’s responses to lineups^24–35^. Most of these studies have relied on observable response rates, such as rates of correct culprit identifications, filler identifications and lineup rejections^27–35^. However, when the aim is to understand how a delay affects the detection of the presence or absence of the culprit and guessing-based selections in lineups, this approach does not yield clear conclusions because changes in the observable response rates may result from different underlying processes. For example, in some studies, the rate of correct culprit identifications from lineups have been found to decline with delays ranging from several minutes to eleven months^27,28,31^. In other studies, no differences were found in the culprit identification rates as a function of delay^29,30,32,33^. With one notable exception^33^, sample sizes in these studies were relatively small, ranging from *N* = 85 to *N* = 210. Therefore, it remains unclear whether delay had indeed no effect on the culprit identification accuracy or whether the relatively small sample sizes resulted in insufficient statistical sensitivity to detect such effects. A third possibility is that the ability to detect the culprit’s presence within the lineup declines, but participants compensate this decline by being more willing to select someone from the lineup based on guessing, resulting in no substantial change in culprit identification rates even after increasing delays. Indeed, in some studies, a descriptive increase in filler identifications in both culprit-present and culprit-absent lineups with increasing delay has been reported^24–26,33^. For instance, in one study^33^ there was no detrimental effect of a 48-h delay on the rate of culprit identifications, but a descriptive increase in filler identifications in both fair culprit-present lineups (from 10 to 15%) and fair culprit-absent lineups (from 24 to 45%). This pattern of results raises the question about whether the ability to detect the culprit’s presence was truly unaffected by the delay or whether participants perhaps compensated for a deficit in culprit-presence detection by being more willing to select someone based on guessing, the latter of which would be consistent with the observation that the rates of filler identifications were increased at a descriptive level. However, in other studies no increase was found in filler identification rates as a function of delay^28,31,32,36^. Also, the effects of delay on innocent-suspect identifications are somewhat mixed. Whereas no such effects have been reported in studies with a designated innocent suspect^26,33^, relatively small effects have been reported in studies without a designated innocent suspect in which, therefore, the innocent-suspect-identification rate could not be computed directly but had to be substituted by a value determined by dividing the number of filler identifications in culprit-absent lineups by the lineup size^24,25^.

In addition to studies focusing on observable response rates, a few studies have used ROC analyses to examine the effects of delay on lineup performance^26,33^. One important limitation of this approach is that information provided by filler identifications is disregarded. Signal-detection-theory-based ROC analyses were not originally designed for analyzing lineup data (but see^37^ for a series of thought experiments on how to incorporate the information provided by filler identifications within a signal-detection-theory-based framework). To fit lineup data into the binary format required by ROC analyses, filler identifications and lineup rejections in culprit-present lineups are treated as a single “false rejection” category and filler identifications and lineup rejections in culprit-absent lineups are treated as a single “correct rejection” category. This data reduction discards important distinctions among response types^38–40^. For instance, filler identifications in culprit-absent lineups are false responses, whereas rejections of culprit-absent lineups are correct responses, indicating that different processes underlie these responses. Another limitation of ROC analyses is that they yield only one single performance metric: the partial area under the curve. While reducing lineup data to a single performance metric may be considered sufficient for determining which of two conditions yields superior overall performance, this approach is limited when the aim is to disentangle the underlying processes that give rise to different lineup responses. For instance, when researchers sought to examine the effect of delay on response bias, they had to abandon the measurement model on which ROC analyses are based. In the studies mentioned above, researchers either reverted to analyzing raw response rates^33^ or used the response bias measure *c* derived from signal detection theory^26^. In each case, this entailed a shift to a different measurement model, grounded in assumptions that differ from those implied by ROC analyses.

**Fig. 1:**
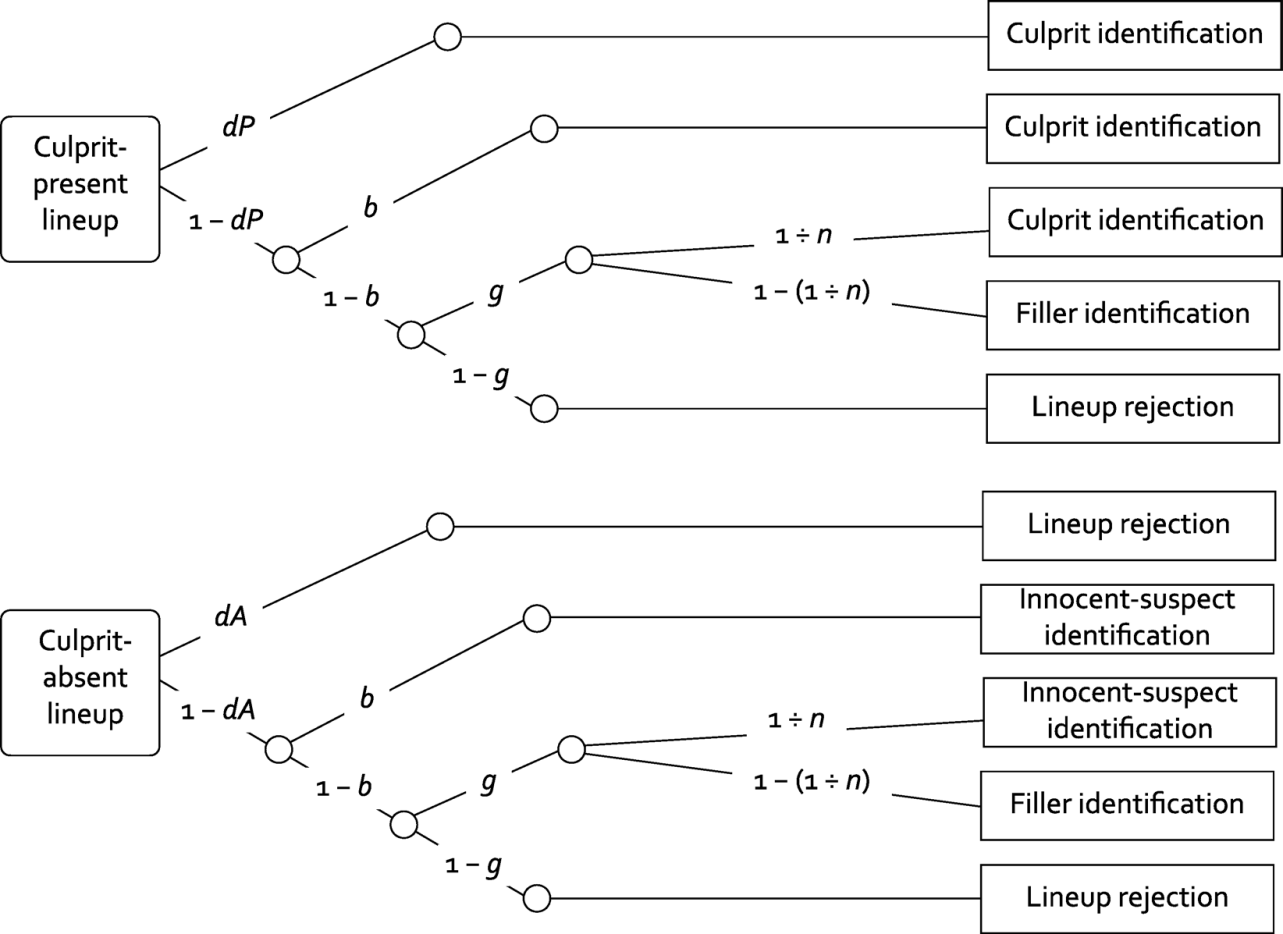
Graphical illustration of the 2-HT eyewitness identification model. Rounded rectangles on the left represent the two types of lineups an eyewitness may be confronted with: culprit-present lineups and culprit-absent lineups. The rectangles on the right represent the categories of observable responses. Letters along the branches denote parameters representing the processes specified by the model: *dP* represents the probability of culprit-presence detection, *b* represents the probability of biased suspect selection which occurs in unfair lineups, *g* represents the probability of guessing-based selection and *dA* represents the probability of culprit-absence detection. The constant 1 ÷ *n* represents the probability of selecting the culprit (upper tree) or the innocent suspect (lower tree) if guessing-based selection occurs, with *n* corresponding to the number of individuals in the lineup.

The present research goals require a comprehensive model specifically designed to separately measure the detection of the culprit’s presence or absence and guessing-based selection in the lineups while taking into account the full pattern of lineup-response categories. Ideally, such a model should be supported by validation studies demonstrating that it reliably captures the processes it was designed to measure. In addition, such a model should allow for a formal evaluation of model fit to the data. Therefore, we used the two-high threshold (2-HT) eyewitness identification model^41–47^, illustrated in Fig. 1. This model serves to trace back lineup responses to distinct underlying processes, which is essential for testing how crime-to-lineup delay affects the detection of the culprit’s presence or absence and guessing-based selection in lineups. Specifically, the 2-HT eyewitness identification model allows for the assessment of four distinct processes within a single, unified framework. These processes, represented by the model’s parameters, are culprit-presence detection, biased suspect selection, guessing-based selection and culprit-absence detection. Parameters are determined based on the complete information drawn from all six categories of observable eyewitness responses to both culprit-present and culprit-absent lineups. In culprit-present lineups, these categories are culprit identifications, filler identifications and lineup rejections; in culprit-absent lineups, they are innocent-suspect identifications, filler identifications and lineup rejections (see the rectangles on the right side of Fig. 1). The model has been thoroughly validated, showing that its parameters reliably and sensitively reflect the processes they were designed to measure. The model’s validity has been demonstrated in a series of experimental studies using the same stimulus materials as those used in the present study^44^, and through reanalyses of published data sets^41^ from various research groups around the world that used diverse lineup procedures, staged-crime videos and photographs of fillers and suspects^32,33,48–53^.

The 2-HT eyewitness identification model belongs to the class of multinomial processing tree models—a class of straightforward and transparent measurement models for which comprehensive tutorials^54^ and easy-to-use software^55^ exist. Multinomial processing tree models are widely used in various domains of cognitive research^54,56–58^. In these models, observable responses are conceived of as being determined by an interplay of different processes that occur with certain probabilities^58^. As explicated previously^41–47^, the probabilities of these processes occurring are represented by model parameters for which estimates can be determined and which can be statistically compared. The processes of culprit-presence detection, biased suspect selection, guessing-based selection and culprit-absence detection are precisely and transparently defined by the model’s structure, as formalized in the model equations and illustrated in Fig. 1. The verbal labels used for the parameters only serve as accessible everyday-language descriptors to simplify communication. The following sections use these labels to explicate the model equations.

In a culprit-present lineup (see the upper tree in Fig. 1), it is assumed that the presence of the culprit is either detected or not detected. This detection-based process may depend, for example, on how clearly or how long the culprit’s face was visible at encoding^41,44^. With probability *dP*, the presence of the culprit is detected, which leads to a correct identification of the culprit. With probability 1 − *dP*, the culprit’s presence is not detected. In this case, the culprit can still be identified through one of two non-detection-based processes: biased selection and guessing-based selection. Biased selection occurs with the conditional probability *b*. This process is determined by the fairness of the lineup^42^. If the lineup is unfair and the suspect noticeably stands out due to physical appearance or distinctive characteristics of the photo, biased suspect selection occurs with the conditional probability *b* > 0. In case of no biased suspect selection, which occurs with the conditional probability 1 − *b*, a lineup member may still be selected based on guessing, which occurs with the conditional probability *g*. Guessing-based selection is defined as a selection process that is neither driven by detection nor by bias, implying that there is no systematic preference for selecting the suspect over the fillers. Specifically, the probability of the selection of the culprit is determined by the sampling probability 1 ÷ *n*, with *n* being a constant representing the number of individuals (suspect and fillers) in the lineup. For example, in a lineup consisting of six individuals, the probability of selecting the culprit as a consequence of a guessing-based process is 1 ÷ 6 = 0.1$\bar{6}$. The probability of selecting a filler as a consequence of a guessing-based process is given by the complementary probability 1−(1 ÷ *n*). In six-person lineups, this probability is 5 ÷ 6 = 0.8$\bar{3}$, illustrating that, as Wixted and Wells^59^ have noted, “a witness who chooses randomly is far more likely to land on a filler than the suspect”^59^. In case of no guessing-based selection, which occurs with the conditional probability 1 − *g*, the lineup is falsely rejected.

The model also includes a second detection-based process for culprit-absent lineups (see the lower tree in Fig. 1). Here, the absence of the culprit is detected with probability *dA*, leading to a correct rejection of the lineup. With probability 1 − *dA*, culprit-absence detection does not occur. In this case, biased suspect selection may occur with the conditional probability *b*, resulting in the selection of the innocent suspect. In case of no biased suspect selection, which occurs with the conditional probability 1 − *b*, a lineup member may still be selected based on guessing, which occurs with the conditional probability *g*. The probability of this guessing-based process leading to the selection of the innocent suspect is determined by the sampling probability 1 ÷ *n*, while the probability of selecting a filler is 1−(1 ÷ *n*). In case of no guessing-based selection, which occurs with the conditional probability 1 − *g*, the lineup is correctly rejected.

To date, the effects of delay on culprit-presence detection, guessing-based selection and culprit-absence detection have not been examined directly, which was therefore the aim of the present study. Furthermore, with a few notable exceptions^29,31,35^, manipulations of the delay variable in previous studies in which lineups were presented were typically limited to only two points in time: one condition with no or a small delay and one condition with a larger delay^24–26,30,32–34^. This binary approach, while certainly informative, does not capture the form of the changes occurring over time. We therefore decided to examine the effects of delay across four points in time with the goal to provide a more comprehensive understanding of how the processes underlying eyewitness responses to lineups change as a function of delay. Specifically, we investigated changes in culprit-presence detection, guessing-based selection and culprit-absence detection as defined within the 2-HT eyewitness identification model with no delay, a delay of one day, a delay of one week and a delay of one month between viewing a staged-crime video and responding to the lineups.

The first prediction about the effects of these delays refers to the memory-based process of culprit-presence detection (parameter *dP*). As memory is susceptible to forgetting^4^, we expected parameter *dP* to decline as a function of delay in the form of a typical forgetting function^16^. In contrast, two different predictions as to how guessing-based selection (parameter *g*) changes as a function of delay were possible based on prior research. The inference for deriving one of the predictions begins by noting that guessing-based selection is known to be very sensitive to the probability with which eyewitnesses expect the culprit to be in the lineup based on the instructions they receive^44,46^. Theses instructions were the same for all delay conditions. Thus, one possible prediction was that guessing-based selection stays constant across delays. Alternatively, the possibility exists that, given a decline in culprit-presence detection as a function of delay, participants might engage in compensatory guessing^60–63^. If this were the case, then the probability of guessing-based selection would increase as a function of increasing delays.

Finally, the prediction about delay-induced changes in culprit-absence detection (parameter *dA*) was not as straightforward. On the one hand, the memory-based process of culprit-absence detection should become less likely with increasing delay, just like the memory-based process of culprit-presence detection. On the other hand, low estimates for *dA* have often been reported in empirical studies even under no-delay conditions^42,45,46,64^ because culprit-absence detection is an inherently demanding process. Whereas culprit-presence detection requires only one lineup member (the culprit) to elicit culprit-presence detection, culprit-absence detection requires the eyewitness to rule out every single lineup member as the culprit. Consequently, while a delay may still negatively affect culprit-absence detection, the to-be-expected low value of *dA* even in the no-delay condition makes further substantial reductions unlikely.

## Method

### Participants

Participants were recruited via the Horizoom research panel (www.horizoom-panel.de), a panel certified under ISO 20252 which ensures rigorous quality control. All participants were first exposed to the staged-crime video and were then randomly assigned to one of four groups defined by the duration of the delay after which the participants were invited to participate in the second phase of the experiment. Of the 3,108 data sets of participants who had given their informed consent before being exposed to a staged-crime video, 22 had to be excluded because participants had not passed the attention check (see below), 31 had to be excluded because of duplicate participation and 245 had to be excluded because participants had not completed the experiment or had withdrawn their consent after having completed the first phase of the experiment. Therefore, valid data sets of 2,810 participants were available after the first phase of the experiment in which participants had been exposed to the staged-crime video. Of these participants, 550 were assigned to the no-delay condition and were asked to respond to the lineups right after having seen the staged-crime video, 788 participants were assigned to the 1-day-delay condition, 706 participants were assigned to the one-week-delay condition and 766 participants were assigned to the 1-month-delay condition. More participants were assigned to the with-delay conditions than to the no-delay condition in an attempt to compensate for anticipated dropouts.

Responses to lineups were collected from 550 participants in the no-delay condition, from 532 participants in the one-day-delay condition, from 520 participants in the 1-week-delay condition and from 506 participants in the 1-month-delay condition, resulting in a total of 2,108 data sets that were analyzed. A sensitivity analysis with G*Power^65^ showed that given *N* = 2,108 participants and four responses per participant, error probabilities of α = β = 0.05 (and thus a power of 0.95) and *df* = 3 for tests of parameter equality across the four delay conditions, effects of delay as small as *w* = 0.05 could be detected (for further details, see https://osf.io/fqus6).

The groups were compared with respect to the demographic data that we had collected to assess whether there was any indication that the dropout was selective. Mean age and age range, gender and educational level are reported in Table 1. Neither age, *F*(3, 2104) = 0.59, *p* = .619, nor gender distribution, χ²(6) = 5.23, *p* = .515, nor educational level, χ²(3) = 4.42, *p* = .220 differed significantly among groups. Thus, even though the sample size and, hence, the sensitivity of the statistical tests of differences among groups was rather high, there was no evidence that the dropout was selective.

**Table 1:** Mean age (standard deviations in parentheses), gender and educational level by delay. A-Levels include international baccalaureate (IB) or equivalent qualifications.

Delay	Mean age (standard deviation)	Gender	Educational level
No delay (*n* = 550)	52 years (15 years)	45% ♀, 55% ♂, < 1% non-binary	55% A-Levels or higher
One day (*n* = 532)	51 years (14 years)	44% ♀, 55% ♂, 1% non-binary	59% A-Levels or higher
One week (*n* = 520)	51 years (14 years)	43% ♀, 57% ♂	60% A-Levels or higher
One month (*n* = 506)	51 years (14 years)	41% ♀, 58% ♂, 1% non-binary	55% A-Levels or higher
All (*n* = 2108)	51 years (14 years)	43% ♀, 56% ♂, < 1% non-binary	57% A-Levels or higher

### Ethics statement

The ethics committee of the Faculty of Mathematics and Natural Sciences at Heinrich Heine University Düsseldorf approved the experiment. The experiment was conducted in accordance with the Declaration of Helsinki. Participants provided informed consent before participating. In the consent form and prior to viewing the staged-crime video, participants were informed that the video would contain physical and verbal violence. They were instructed to proceed with the study only if they were comfortable watching such content.

### Materials and procedure

Materials and procedure were the same as those used in a number of previous studies^41–47,66^ except for the manipulation of the delay between viewing the staged-crime video and responding to the lineups. The experiment was conducted online using *SoSci Survey*^67^ (www.soscisurvey.de). Participation was possible with a desktop or a laptop computer. Participants were informed that the experiment consisted of two parts, the first of which they would complete directly. In addition, participants were informed that the experiment would include a video portraying physical and verbal violence. They were advised that participation required their consent to view such a video and to the use of their data. Next, participants provided their age, gender and educational level. Participants could participate only if they were least 18 years old (a legal requirement in Germany).

After having been instructed to start the video by clicking on a “Start“ button, each participant watched one of two staged-crime videos (referred to as Video 1 and Video 2). The videos were presented at a resolution of 885 × 500 pixels and lasted approximately 130 seconds. The videos depicted the same events in the same sequence and timing but the actors differed between the videos. However, the actors playing the same characters in both videos were chosen to be similar in body shape, hair color and hairstyle. For instance, the actor playing Character A in Video 1 resembled the actor playing Character A in Video 2. The same applied to Characters B, C and D. Both videos featured four men dressed in FC Bayern München soccer club fan clothing who physically and verbally assaulted a man in Borussia Dortmund fan clothing at a bus stop. All culprits were involved in the crime to a similar extent.

By including four culprits, we were able to obtain four data points per participant, thereby increasing the statistical sensitivity of our analyses while also maintaining ecological validity given that more than one third of real-world crimes have been reported to involve multiple culprits^68^. Both in the real world and in study settings, responding to multiple lineups after having witnessed a multiple-culprit crime may be more cognitively demanding than responding to a single lineup after having witnessed a single-culprit crime^69^.

Following the video, participants answered an attention-check question in which they had to identify the roles of the protagonists in the video. The correct response was to select “soccer fans” among nine distractor options such as “dancers”, “farmers” or “artists”. Providing a correct response to the attention-check question was a prerequisite for participation in the second part of the experiment.

Participants assigned to the no-delay condition were then informed that they were about to enter the second part of the experiment. Participants assigned to one of the with-delay conditions were instead informed that they would receive an email inviting them to participate in the second part of the experiment after a delay of 24 h (1-day-delay condition), 7 days (1-week-delay condition) or 30 days (1-month-delay condition). Participants in the with-delay conditions did not always complete the second part of the experiment after the nominal delay, that is, on the same day at which the invitation email had been sent. The average actual delay was therefore somewhat larger than the nominal delay. The average actual delay was 1 day (standard deviation < 1 day) in the 1-day-delay condition, 8 days (standard deviation = 1 day) in the 1-week-delay condition and 33 days (standard deviation = 3 days) in the 1-month-delay condition.

In the second part of the experiment, participants were instructed to identify the FC Bayern München fans—seen in the video during the first part of the experiment—from a series of photo lineups. The following instructions were given (the original instructions were in German):

In the first part of the experiment, you saw a film with Bayern München fans. Now we want you to identify them. To do this, we will show you several lineups. In each lineup, you will see a series of faces. You will be asked to indicate whether one of the people in the lineup is one of the Bayern München fans. It is also possible that no one in the lineup is one of the Bayern München fans. If you recognize someone, click on the ‘Yes, was present’ button that belongs to the recognized face. Otherwise, click on the ‘No, none of these persons was present’ button.

Afterwards, each participant was presented with four separate lineups, one for each of the Bayern München fans from the video. In each lineup, one suspect and five fillers were displayed simultaneously in a single row. This presentation format is a possible method for photographic lineups^70–74^ and was chosen for several reasons. First, it resembles the arrangement used in in-person lineups, which remain part of the pertinent guidelines in various jurisdictions^75,76^. Second, it has been reported that, within these guidelines, “52% described an identification procedure that suggested lineup members would be presented simultaneously (e.g., they would appear in a *line*)”, (emphasis added^75^, p. 302). Based on this, we considered the single-row photographic format to be a reasonable choice for the present study.

As in earlier studies^41–47,66^, the crossed-lineup procedure was used. Two of the four lineups were culprit-present lineups and two were culprit-absent lineups. The two culprits for the culprit-present lineups were selected randomly without replacement from the four culprits of the video the participant had seen. The innocent suspects in the culprit-absent lineups were culprits from the parallel video that the participant had not seen. For example, if Characters C and D from Video 1 had randomly been selected as culprits in the culprit-present lineups, then Characters A and B from Video 2 were selected as innocent suspects in the culprit-absent lineups. This crossed-lineup procedure ensures that the photos of the culprits and innocent suspects (taken right after the videos had been recorded) differ to the same degree from the photos of the fillers which were taken from a face database^77^ and resembled one of the culprits in body shape, hair color and hairstyle. This setup is analogous to real-world situations where photos of suspects (whose guilt or innocence is unknown to the police) often come from a different source, such as social media, than the filler photos which are typically taken from a face database. The crossed-lineup procedure is similar, but not identical, to the single-lineup procedure proposed by Oriet and Fitzgerald^78^. In the single-lineup procedure, a single lineup is shown to all participants and the suspect’s guilt is determined by which of two videos participants viewed (either featuring the suspect from the lineup or a similar-looking person). As suggested by Quigley-McBride and Wells^79^ it is advisable to additionally determine randomly for each participant whether a suspect appears in a culprit-present or culprit-absent lineup. This randomization ensures that each suspect has an equal likelihood of being presented as a culprit or as an innocent suspect (see^80^, for an early implementation of this technique). This makes the crossed-lineup procedure particularly suitable for cases involving multiple culprits, as it allows for variation across lineups in whether a culprit or an innocent suspect is presented.

All individuals were shown from a frontal view with neutral facial expressions against a black background with no visible clothing. The photographs were adjusted to maintain consistent face sizes and lighting conditions and were displayed at a resolution of 142 × 214 pixels. The positions of the photos in the lineups were determined randomly, as was the sequence of the lineups. Once participants had responded to all lineups, they were asked to reaffirm, or to withdraw, their consent to the use of their data. They were then debriefed, thanked for their participation and redirected to the panel provider to receive their monetary compensation.

## Results

The response frequencies obtained in this experiment are presented in Table 2. The files with the raw frequency data and the equations needed for the model-based analyses are available at https://osf.io/fqus6.

**Table 2:** Response frequencies (and proportions, relative to the condition-specific response frequencies in culprit-present and culprit-absence lineups, respectively, in parentheses) as a function of culprit presence or absence and delay.

Delay	Culprit-present lineups			Culprit-absent lineups		
	Culprit identifications	Filler identifications	Lineup rejections	Innocent-suspect identifications	Filler identifications	Lineup rejections
No delay	401 (37%)	276 (25%)	423 (38%)	133 (12%)	383 (35%)	584 (53%)
One day	265 (25%)	355 (33%)	444 (42%)	137 (13%)	397 (37%)	530 (50%)
One week	188 (18%)	359 (35%)	493 (47%)	117 (11%)	396 (38%)	527 (51%)
One month	134 (13%)	347 (34%)	531 (52%)	109 (11%)	370 (37%)	533 (53%)

All model-based analyses were conducted using *multiTree*^55^. Four instances of the model illustrated in Fig. 1 were needed to analyze the data, one instance for each delay condition (no delay, 1 day, 1 week, 1 month). To generate a testable base model, restrictions were applied to the 2-HT eyewitness identification model. As six-person lineups were presented in the current experiment, the term 1 ÷ *n* which represents the sampling probability of the suspect in case of guessing-based selection was set to 0.16667 for all conditions. Given that the lineups consisted of the same suspects and fillers in all conditions, there was no reason to expect differences in lineup fairness among conditions. Consequently, parameter *b* was set to be equal for all conditions. As shown by the goodness-of-fit statistic *G*^2^ which is asymptotically chi-square distributed with degrees of freedom indicated in parentheses (see^81^ for details), the base model incorporating these restrictions fit the data, *G*^2^(3) = 2.87, *p* = .412, supporting the conclusion that lineup fairness, represented by parameter *b*, did not vary as a function of delay. This implies that the lineups were equally fair across all delays. Parameter *b* was estimated to be 0.04 (95% CI [0.03, 0.06]), reflecting a slight inherent unfairness in the lineups across all delays. By taking biased selection due to lineup unfairness into account explicitly in the 2-HT eyewitness identification model, two important goals are achieved. First, the model provides for a direct measure of lineup fairness that is more valid than measures based on the traditional mock-witness task because the processes involved differ between mock witnesses and eyewitnesses^42^. Second, the model also ensures that the measurement of the other model parameters remains uncontaminated by lineup unfairness^41,42,44^.

**Figure 2.**
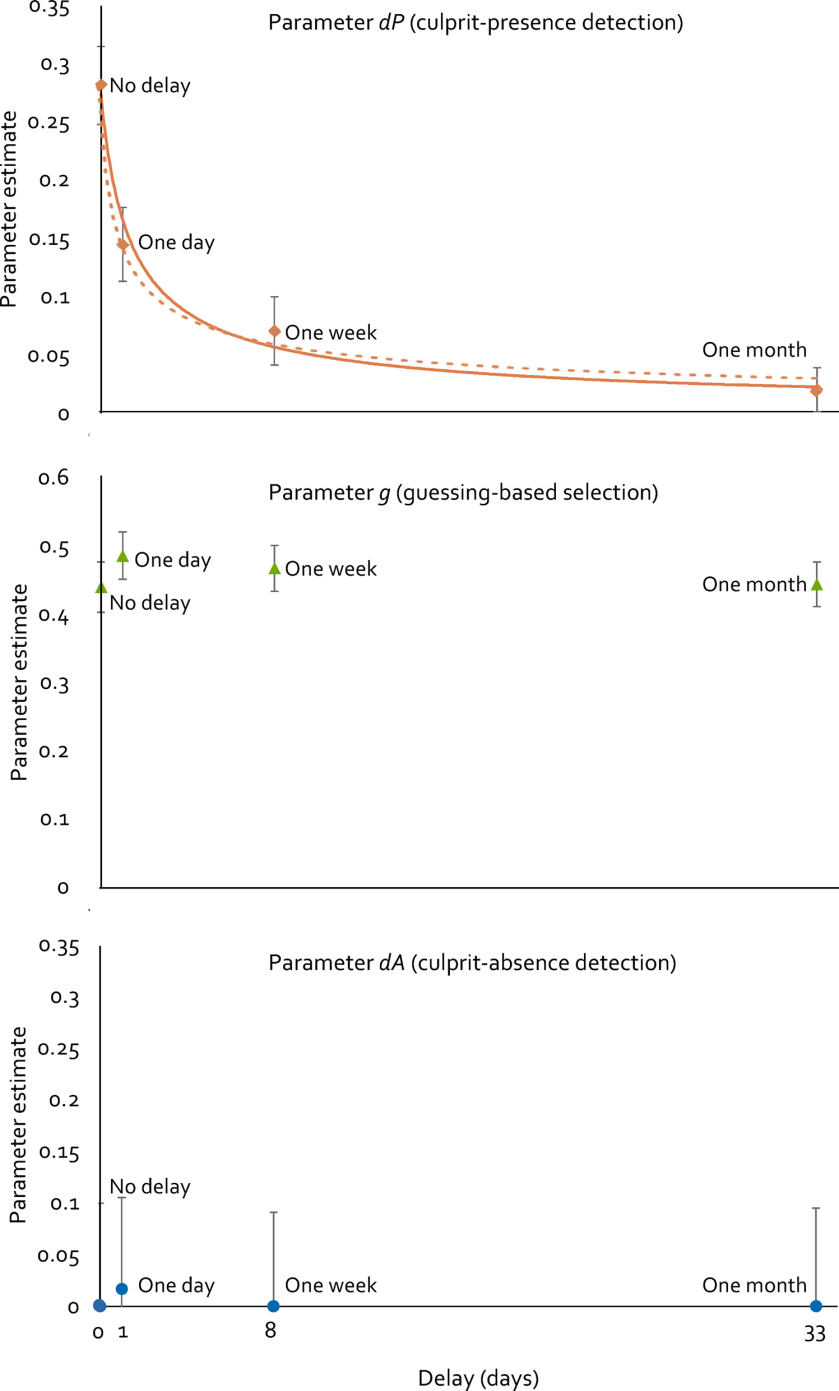
2-HT eyewitness identification parameter estimates as a function of the average actual delay. For the no-delay condition and the one-day-delay condition, the nominal delay was equal to the average actual delay. For the condition with a nominal one-week delay, the average actual delay was eight days. For the condition with a nominal delay of 1 month, the average actual delay was 33 days. Estimates of parameter *dP* (culprit-presence detection) are shown in the upper panel together with graphical illustrations of two functions that have been fitted to the estimates of parameter *dP*. The continuous orange curve shows the standard power function, the dashed orange curve shows the simplified function derived from Wickelgren’s^82^ power-exponential forgetting theory by Wixted and Carpenter^83^ (see text for details). Both curves are plotted so as to align with the empirical data points on the delay axis (0, 1, 8, 33 days). Estimates of parameter *g* (guessing-based selection) are shown in the middle panel. Estimates of parameter *dA* (culprit-absence detection) are shown in the lower panel. Error bars represent 95% confidence intervals.

The estimates of parameters *dP* (culprit-presence detection), *g* (guessing-based selection) and *dA* (culprit-absence detection) are displayed in Fig. 2. The estimates of parameter *dP* (upper panel of Fig. 2) clearly decline as a function of delay. To test whether this decline is statistically significant, parameter *dP* was set to be equal across all four delay conditions. The reduction in fit of the model with this equality restriction relative to the base model was statistically significant, Δ*G*^2^(3) = 176.43, *p* < .001. The model implying that parameter *dP* does not differ among delay conditions thus must be rejected, leading to the conclusion that the probability of culprit-presence detection declines as a function of delay. Next, the two most successful of the five ‘classic’ functions describing forgetting curves^4^, a power function and a logarithmic function, were fitted to the estimates of parameter *dP*. Here we took into account that the standard power and logarithmic functions, *dP* = λ · (*delay*)^−ψ^ and *dP* = λ · ln(*delay*) + ψ, are undefined at *delay* = 0. In doing so we followed Wixted and Ebbesen^16^ and used modified versions of these standard functions of the form *dP* = λ · (1 + *delay*)^−ψ^ and *dP* = λ · ln(1 + *delay*) + ψ, which are defined at *delay* = 0 and quickly approximate *dP* = λ · (*delay*)^−ψ^ and *dP* = λ · ln(*delay*) + ψ, respectively, as delay increases. Both functions fit the data well, but the best fitting power function, $\it \:\widehat{\text{dP}}$ = 0.2744 · (1 + *delay*)^−0.726^, *R*^2^ = 0.98 (shown as the continuous orange curve in Fig. 2) fit the data even better than the best fitting logarithmic function, $\it \:\widehat{\text{dP}}$ = −0.0679 · ln(1 + *delay*) + 0.2377, *R*^2^ = 0.88 (not shown in Fig. 2). Whereas these functions provide an excellent description of the data, they are not theoretically motivated. The latter approach—to fit a theoretically motivated forgetting function—was taken in a meta-analysis by Deffenbacher et al.^18^ who fit the function implied by the power-exponential forgetting theory proposed by Wickelgren [e. g.,^82^] to eleven data sets obtained in facial memory studies. However, while Deffenbacher et al.^18^ noted that this function was of necessity only fitted by eye due to limited data points making formal data fitting impractical^18^, they also discussed the simplified version of Wickelgren’s power-exponential forgetting function^82^ proposed by Wixted and Carpenter^83^. This simplification makes this forgetting function more practical for data fitting in empirical memory studies. Specifically, Wixted and Carpenter^83^ have shown that, under typical conditions, Wickelgren’s power-exponential forgetting function reduces to

1 $$m\,=\,\lambda {({\text{1}}\,+\,\beta t)^{ - \psi }},$$

where *m* is memory strength, λ is the state of long-term memory at *t* = 0, β is a scaling parameter, *t* is the time delay and ψ is the rate of forgetting. This function can be fitted to the present data given a boundary condition proposed by Wickelgren^82^. This boundary condition is that *m*(*t* = 0) = λ, which was therefore equated with *dP*(*t* = 0). An estimate of *dP* at *t* = 0 is known ($\it \:\widehat{\text{dP}}$ = 0.2820 in the immediate condition, see Fig. 2). It is therefore straightforward to set λ = 0. 2820. Given this, the fit of the simplified version of the forgetting function implied by Wickelgren’s power-exponential forgetting theory^63,64^ is excellent ($\it \:\widehat{\text{dP}}$ = 0.2820 · (1 + 2.6623 · *delay*)^−0.502^, *R*^2^ = 0.99) and is shown as the dashed orange curve in Fig. 2. In the light of the similarly excellent fit of the descriptive power function mentioned above, the fact that this theoretically motivated forgetting function fits the present data so well may not be too surprising as it is, after all, also a power function, the only difference to the standard power function being the additional scaling parameter β in the theoretically motivated forgetting function.

The estimates of parameter *g*, in contrast, seem to be relatively constant across delays (middle panel of Fig. 2). To test whether this is indeed the case, parameter *g* was set to be equal across all four delay conditions. The reduction in fit of the model with this equality restriction relative to the base model was not statistically significant, Δ*G*^2^(3) = 6.26, *p* = .100. The model implying that parameter *g* does not differ among delay conditions is compatible with the data and need not be rejected, leading to the conclusion that the probability of guessing-based selection does not significantly change as a function of delay.

Finally, the estimates of parameter *dA* were very low (bottom panel of Fig. 2). Given that the parameter estimates were so close to the boundary of the parameter space, we used the parametric bootstrap procedure implemented in multiTree^55^ to obtain a *p-* value based on a simulated sampling distribution^54,84^. The model in which parameter *dA* was set to be equal across all four delay conditions did not fit significantly worse than the base model, Δ*G*^2^(3) = 0.14, bootstrapped *p* =. 814, indicating that the probability of culprit-absence detection does not significantly change as a function of delay.

## Discussion

The goal of the present study was to examine how the processes underlying eyewitness responses are affected by the delay between viewing a staged-crime video and responding to lineups. More specifically, we tested hypotheses about how delays affect culprit-presence detection, guessing-based selection and culprit-absence detection. Extending previous studies in which the effects of delay were typically investigated by comparing a condition with no or a small delay to another condition with a larger delay^24–26,30,32–34^, here the delay variable had four levels: no delay, one day, one week (average actual delay: eight days) and one month (average actual delay: 33 days), allowing us to examine the form of changes in culprit-presence detection, guessing-based selection and culprit-absence detection over time. This was done using a large sample of *N* = 2,108 participants, each of whom contributed four data points, thus providing for sensitive statistical tests of the effects of delay on the processes underlying eyewitness responses to lineups.

The results are clear-cut for the memory-based process of culprit-presence detection (parameter *dP*) which declines in a way that is described well by a power function, one of the two ‘classic’ functions that have been found to best describe the decline in the ability to remember over time^4^. In fact, the present results are strikingly parallel to those from experimental paradigms as diverse as human face memory, matching-to-sample with pigeons and even Ebbinghaus’ original savings data for which a power function has been found to fit forgetting curves best and even slightly better than a logarithmic function, just like in the present case^16^. Additionally, the present results align closely with the simplified version of Wickelgren’s power-exponential forgetting function [e. g., 82] proposed by Wixted and Carpenter^83^. In sum, then, the changes in the memory-based process of culprit-presence detection (parameter *dP*) as a function of delay are consistent with what is known about the time-course of forgetting in general. With four levels of the delay variable, the study presented here allows for this conclusion which would not have been possible to draw based on only two levels of the delay variable, the latter of which is characteristic of most studies on the effects of delay on eyewitness memory^24–26,30,32–34^.

In recommendations of how to perform lineups it has been noted that “eyewitness memory can fade with the passage of time. Hence, a lineup should be conducted as soon as possible after establishing evidence-based suspicion”^1^. The present results demonstrate just how rapid the fading of memory-based culprit-presence detection can be at small delays already. This underscores the critical importance of conducting lineups as soon as possible after a crime to maximize the chances of culprit identifications based on memory. It also highlights the importance of educating those involved in criminal trials such as jurors about the rapid reduction in memory-based culprit-detection processes within the first days following the crime^85^, particularly when considering that naïve metacognitive judgements typically do not anticipate the rapid declines reflected in empirical forgetting curves^86,87^.

The results are also clear-cut for guessing-based selection (parameter *g*) which does not change as a function of delay. Specifically, guessing-based selection does not increase parallel to the delay-related reduction in culprit-presence detection; thus, there is no evidence of compensatory guessing^60–63^. Culprit-absence detection (parameter *dA*) did not vary as a function of delay. Notably, the value of *dA* was already close to zero in the no-delay condition and remained at this low level across all delays. This pattern of *dA* is commonly observed in the literature^42,45,46,64^ and the explanation for this pattern is straightforward. Whereas culprit-presence detection requires just one lineup member (the culprit) to elicit culprit-presence detection, culprit-absence detection requires the eyewitness to rule out every single lineup member as the culprit which is usually much more difficult.

The present results align with those of previous studies according to which delay primarily affects culprit-present lineups as opposed to culprit-absent lineups^27–29,36^. Consistent with these findings, the most striking descriptive observation at the level of observable behavior is that culprit identification rates decrease with increasing delay (Table 2). A priori, this pattern could have been attributed to various underlying processes, such as a decline in culprit-presence detection, changes in guessing-based selection, or a combination of these and other processes. The model-based analysis presented here disambiguates this pattern by demonstrating that the decrease in culprit identification rates is driven by a pronounced decline in culprit-presence detection as a function of delay. By contrast, guessing-based selection and all other processes remain constant across delays.

As a limitation, it should be mentioned that the forgetting functions evaluated here describe the decline in culprit-presence detection but do not allow conclusions about mechanisms of forgetting such as decay, interference or consolidation. Future studies could aim at further disentangling the contributions of these mechanisms. Furthermore, the present conclusions rely on a single experiment, albeit one with a particularly large sample size, to assess the effects of four levels of delay (no delay, one day, one week, one month) on the processes underlying eyewitness responses. While the present results are consistent with the conclusions from previous research indicating that the decline of memory can be described by a power function^16^, future research could further test the robustness of the conclusions drawn here by investigating even longer delays and sequential lineups versus simultaneous lineups, as well as other variations in lineup procedures. Furthermore, while the 2-HT eyewitness identification model has been validated extensively with data from diverse research groups, stimulus materials and lineup procedures^41^, it may be seen as a limitation that, in the present study, the same stimulus materials and lineups as in previous studies^41–47,66^ were used. Replications with different stimulus materials and single-culprit staged-crime videos [e. g.,^88^] could be done to examine generalizability. Another limitation of the present study is that it was not designed to perform formal comparisons across different frameworks for analyzing lineup data. Accordingly, our conclusions are confined to the specific hypotheses tested regarding the effects of delay on culprit-presence detection, guessing-based selection and culprit-absence detection.

In sum, the results of the present study underscore the critical importance of conducting lineups as soon as possible after a crime to enhance memory-based culprit-presence detection. Archival studies from Great Britain suggest that the most common delay between a crime and an associated lineup is one to three months^20–23^. Although it is not justified to generalize the exact time course of the decline in culprit-presence detection observed here to real-world cases, the rapid initial reduction in memory-based culprit detections strengthens the argument that lineups should be conducted as soon as possible, ideally within hours or days after the crime, rather than weeks or months later. These findings also highlight the need to educate jurors and others involved in criminal trials about the sharp decline in memory-based culprit-presence detection within the first days after the crime.

## Data Availability

The files with the frequency data and the equations needed for the model-based analyses are available at https://osf.io/fqus6.
